# Viewpoint on Milestones for Fellowship Training in Movement Disorders

**DOI:** 10.1002/mds.29146

**Published:** 2022-07-11

**Authors:** Jeffrey B. Ratliff, Sara M. Schaefer, Shilpa Chitnis, Jeffrey W. Cooney, Christopher W. Hess, Njideka Okubadejo, Ali Shalash, Elena Moro, Carolyn Sue, Sanjay Pandey, Pramod K. Pal, Laurice Yang

**Affiliations:** ^1^ Department of Neurology Thomas Jefferson University Philadelphia Pennsylvania USA; ^2^ Department of Neurology Yale University New Haven Connecticut USA; ^3^ Department of Neurology University of Texas, Southwestern Medical Center Dallas Texas USA; ^4^ Department of Neurology Duke University Durham North Carolina USA; ^5^ Department of Neurology University of Florida Gainesville Florida USA; ^6^ College of Medicine University of Lagos Lagos Nigeria; ^7^ Department of Neurology, Faculty of Medicine Ain Shams University Cairo Egypt; ^8^ Grenoble Alpes University CHU de Grenoble, Division of Neurology, Grenoble Institute of Neurosciences Grenoble France; ^9^ Department of Neurology Royal North Shore Hospital and Kolling Institute, University of Sydney Sydney New South Wales Australia; ^10^ Department of Neurology Govind Ballabh Pant Institute of Postgraduate Medical Education and Research New Delhi India; ^11^ Department of Neurology National Institute of Mental Health and Neurosciences (NIMHANS) Bengaluru Karnataka India; ^12^ Department of Neurology Stanford University Palo Alto California USA

**Keywords:** Fellowship, Education, Movement Disorders Training, Milestones

There is a demand for quality Parkinson's Disease (PD) care globally and competent movement disorders specialists are needed.^1^, ^2^, ^3^ Fellowship training provides neurologists (residency graduates) education in the diagnosis and management of movement disorders. Graduates may provide improved care for patients with movement disorders, serve as a referral for colleagues, become educators or leaders in the field, and may have scholarly experience during training that prepares them to contribute to the understanding of movement disorders. In North America, there are at least 88 fellowship positions in 2022, compared to 43 positions in 2012.^4^, ^5^ Detailed data outlining the landscape of movement disorders training across global regions is unavailable, but training programs exist in countries across all populated continents. Most programs offer clinical training, research, and didactic conferences, but there is curricular variability.^5^, ^6^ Data suggests improvement in training is needed.^1^

A curriculum developed by the American Academy of Neurology (AAN) Movement Disorders section and the Movement Disorder Society's Pan American Section (MDS‐PAS) outlines content, but provides little methodological guidance for evaluating trainees (Supplementary S1). Other international neurological societies (ie, the MDS, the European Academy of Neurology) financially support clinical or research fellowships in movement disorders ranging from 3 to 12 months at selected centers. There is no established common curriculum for these fellowships. Firmer assessment guidance will improve trainees' clinical competency development.

Milestones are used in medical education internationally, although the definition of “milestone,” differs between regions.^7^, ^8^ Our use of “milestones” adheres to the United States (US)‐based definition, in which milestones are “behaviors associated with a specific level of achievement for [a] competency.”^7^ American physician training programs accredited by the Accreditation Council for Graduate Medical Education (ACGME) use milestones to address inter‐program variability and as a tool to evaluate trainees' growth.^9^ These milestones are not requirements, but provide discrete levels of growing competence trainees can demonstrate via observable behaviors.^10^

## Current Gaps

There are no published competency‐based tools to guide the assessment of movement disorders trainees. Movement disorders fellowships are not regulated or accredited by agencies like the ACGME. Therefore, fellowship educators lack external structure to guide their trainee assessment.

Without periodic evaluation of whether they are meeting goals, curricula drift from their original structure.^11^, ^12^ Revision is challenging without clear metrics to which educators can aspire. The evaluation metrics for a training program are myriad, but the competency of its graduates is paramount.

Fellowship directors may seek resources to build curricula. An available set of milestones specific to movement disorders training reduces the need to “reinvent the wheel” in trainee assessment. Traditionally, fellow assessment has comprised an impression of perceived strengths and weaknesses determined by the fellowship director with the input of collaborating faculty (Supplementary S1). Although this may work for many, the competencies of graduates may vary without uniform benchmarks. Here, we propose and discuss a set of milestones to be adopted for use in clinical training programs by movement disorders educators.

## Methods

An initial set of milestones was created by L.Y. for her trainees. It was presented to the AAN Movement Disorders Subsection Steering Committee and approved for sharing with the subsection during the 2018 AAN Annual Meeting. In March 2019, a fellowship curriculum workgroup formed on AAN and MDS online forums seeking interested educators. The initial workgroup teleconference occurred in April 2019. A sub‐group tasked to create a set of milestones met in May 2019. The process began with the milestones shared at the 2018 AAN meeting. Milestones written by S.S. and J.R. for their respective fellowship programs were incorporated. The group was unaware of other milestone‐based tools in use at other training programs.

The following goals were established: (1) provide a tool for fellowship directors globally to progressively evaluate fellows' performance; (2) avoid mandating training standards while providing an assessment tool that educators can build on for their own program. An initial draft of the milestones was made by July 2019. Ongoing revisions occurred over the year 2020 to 2021 via email and teleconference.

An initial set of milestones was submitted to the journal *Movement Disorders* in February 2021. Recommended revisions included (1) improve alignment with ACGME core competencies; (2) gather input from international movement disorder educators, improving the generalizability to global trainees; and (3) gather feedback from North American fellowship directors.

Subsequent meetings mapped the milestones with the established competencies: Patient Care, Medical Knowledge, Practice Based Learning and Improvement, Systems Based Practice, Professionalism, and Interpersonal Communication (Table 1).^13^

**TABLE 1:** Core competencies and enclosed milestones

**Patient Care**
History Taking
Movement Disorders Examination
Movement Disorders Formulation
Parkinson's Disease (PD)
Other Parkinsonian Disorders
Tremor
Dystonia
Other Hyperkinetic Disorders
Ataxia
Huntington's Disease (HD) and other Choreas
Functional Movement Disorders (FMD)
Therapeutic Chemodenervation
Deep Brain Stimulation (DBS) Programming
**Medical Knowledge**
Anatomy, Neurochemistry, Neurophysiology of Movement Disorders
**Systems Based Practice**
System Navigation for Patient‐Centered Care
**Practice Based Learning** **and Improvement**
Evidence‐Based and Informed Practice
Self‐Directed Learning
Scholarly Work^a^
**Professionalism**
Departmental Accountability and Contribution
Clinical Accountability/Conscientiousness
**Interpersonal and Communication Skills**
Communication within Team
Communication with Patient and Family
Communication with Other Providers

^a^ Scholarly Work is included as an optional milestone for training programs with a scholarship requirement.

In 2021, the Neurology Milestones 2.0 were officially put into use.^14^, ^15^ Notably, this version omitted sub‐specialty specific milestones for residency trainees (eg, epilepsy, movement disorders, etc.). The authors herein decided that, because of the sub‐specialty nature of fellowship training, disease‐based milestones still facilitate educators' assessment of clinical breadth in a program and identification of skill gaps in trainees.

Additional international collaboration was sought. Requests went to multiple individuals, including MDS regional section chairs, leaders on the MDS Education Committee, and those with education leadership expertise in training movement disorders clinicians. Some requests were not returned. In autumn 2021, input from 7 non‐US collaborators was incorporated into the milestones. Some contributors, while contributing to the milestones, excused themselves from the authorship process (see Acknowledgments).

Non‐US collaborators largely agreed with the milestones developed to date, suggesting that trainees' competency development is similar across countries. Key revisions included augmented consideration of systemic diseases (eg, Wilson's), inclusion of general neurologic and medical examination skills in the assessment of movement disorders (eg, ophthalmologic examination), and increased emphasis on genetic history gathering. Minor revisions refined where skills occurred across the levels of each milestone. Group consensus was reached for each edit.

Revised milestones were distributed to the online fellowship directors' forum for commentary and suggestions beyond the workgroup. No suggestions were volunteered. The group agreed on the proposed milestones in May 2022 (Supplementary S2).

## General Features of the Movement Disorders Fellowship Milestones

The Movement Disorders Fellowship Milestones provide a progressive set of skills to assess periodically based on observation of a trainee. The formatting and structure matches the Neurology Milestones.^14^, ^16^ Level 1 represents competencies imbued by a neurology residency or those learned very early in fellowship training. Level 2 and Level 3 reflect skills progressing from simple to complex management. Level 4 delineates competency for independent practice typically achieved on completing fellowship. Level 5 is an aspirational level for fellows who achieve expertise or exquisite skill in a specific domain.

### Patient Care

Milestones mapped to Patient Care (PC) include those critical to the assessment and diagnosis of movement disorders: history taking, movement disorders examination, and formulation. In addition, they include disease‐ and procedure‐specific milestones: Parkinson's disease, other parkinsonian disorders, tremor, dystonia, other hyperkinetic disorders, ataxia, functional movement disorders, therapeutic chemodenervation, and deep brain stimulation (DBS) programming. These disease‐ and procedure‐specific milestones outline observable behaviors demonstrating progressive ability to diagnose and manage their respective disease or apply their respective procedural skill.

Huntington's Disease (HD) and other Choreas are included. Few fellowship programs may house designated HD centers, which limits trainees' clinical exposure. However, the evaluation, diagnosis, and initial management of HD and choreiform disorders are a necessary competency for movement disorder specialists, even those outside a designated HD center.

### Medical Knowledge and Systems‐Based Practice

A single milestone each is mapped to the Medical Knowledge (MK) and Systems‐Based Practice (SBP) competencies, respectively. The former monitors trainees' demonstration of knowledge and familiarity of the anatomy, neurochemistry, and neurophysiology of movement disorders. The latter measures trainees' ability to oversee patients' navigation of their local healthcare system.

### Practice‐Based Learning and Improvement

There are three milestones mapped to Practice Based Learning and Improvement (PBLI). Similar to the Neurology Milestones, these milestones assess the ability to use data and evidence in clinical practice, develop a learning plan, and engage in scholarly activity. The milestone on scholarly activity is listed as optional. Although not all fellowships require scholarship of their trainees, 100% of North American programs have fellows engage in some clinical research.^5^ We believe the ability to assess trainees' scholarly pursuits is important to many programs.

### Professionalism

Two milestones are mapped to Professionalism. They track trainees' interactions among members of the movement disorders team and their professional dedication to their patients.

### Interpersonal and Communication Skills

Three milestones mapped to Interpersonal and Communication Skills (ICS) assess fellows' ability to communicate appropriately with members of the movement disorders team, patients and families, and other healthcare providers, respectively.

## Discussion

The Movement Disorders Fellowship Milestones fill a gap in the educational paradigm of fellowship training and have multiple applications. Primarily, they are a tool for assessment. Milestone incorporation into an individualized assessment program is at the discretion of fellowship directors and educators. These educators are the arbiters of whether a trainee is adequately prepared based on the curriculum completed and the available assessment data. For example, fellowship faculty with expertise in DBS may be best equipped to inform the assessment of trainees in DBS and to provide this assessment data to the program's director. The milestones provide a tool to be used periodically throughout training to assess fellows, but need not be used as a requirement for graduation. Like the Neurology Milestones' recommendation of biannual assessment of trainees, we also recommend periodic evaluation to monitor progress and growth of learners.^14^, ^15^, ^16^ However, we do not dictate the frequency at which adopters should apply these milestones. Many accredited programs that use milestones do so via Clinical Competency Committees (CCC) that interprets assessment data to evaluate learners' progression. Exploring the implementation of CCC's in movement disorders training is an area of possible future education research.

The milestones can help educators worldwide to develop and refine curricula. Many movement disorders fellowships report having a standardized extra‐clinical curriculum to supplement the clinical experience.^5^ If educators find that fellows struggle to achieve mastery in certain milestone competencies, curricula can be augmented in a timely manner with additional clinical or didactic experiences. At the residency level, this practice is applied to plan for curricular development and improvement.^17^

The milestones aid remediation by helping educators identify gaps among individual trainees. For trainees, the milestones increase transparency regarding the expected skills to develop. This helps trainees identify their own clinical skill gaps, therefore, providing an aid to help focus their learning and clinical training. Educators can adjust curricula rapidly to allow fellows to rotate in clinical domains in which they need improvement over those they have already mastered. Through curriculum refinement and targeted remediation, the clinical quality of sub‐specialists joining the field increases.

As with other milestone models, there is no timeline along which fellows must attain certain levels. Although the milestones likely best fit the common 2‐year fellowship timeline seen in North America, there are movement disorders training programs as brief as 3 months in Europe. The intention of these milestones is not to impose curricular uniformity across all programs, but to provide a tool that programs could apply to their educational needs. The milestones may be useful in shorter programs, although educators may acknowledge that developing clinical mastery in the management of less common movement disorders may be more challenging. Approximately 60% to 70% of all patient encounters in a fellowship program are dedicated to parkinsonism.^6^ Therefore, fellows may advance rapidly through milestones related to parkinsonian diseases. Given the variety in movement disorders training models, learners' progress through these milestones may vary. This variety is a possible area of future study, especially in comparing training programs of differing duration.

Fellows' clinical training depends on factors like geography, institutional resources, and clinical specialization of their faculty. In some countries, not all resources are available (ie, invasive PD treatment, botulinum toxin, and single‐photon emission computed tomography [SPECT] imaging), and therefore, a fellowship might choose applicable milestones accordingly.^18^ There are other clinical skills not captured in these milestones; they are not exhaustive. For example, the group considered the inclusion of intraoperative monitoring skills for movement disorder functional neurosurgery. Some programs have the expertise and facilities to train fellows in other electrophysiological methods used in the diagnosis and management of movement disorders. Another example of an omitted clinical domain is movement disorder emergencies, which we felt was a rare event typically evaluated by non‐fellowship trained hospital‐based neurologists, making observation of demonstrable skills in movement fellows challenging. Overall, expanding the milestones further would likely detract from their simplicity, prove overly cumbersome to potential assessors, and make adoption less likely. We hope their adoption inspires educators that train fellows in omitted domains, to think critically about the path their trainees follow to clinical competency.

These milestones were developed via expert opinion among movement disorders educators from multiple countries, originating from an online forum open to educators in North America with original volunteers exclusively from the United States. The authorship represents a diversity of countries, but perspective from all global regions is lacking. Therefore, potential biases may exist in these milestones and we hope that education researchers across the globe will adopt these milestones and share their experiences in the movement disorders education literature.

Dissemination and implementation of the milestones is only a first step in educational reform. The milestones provide educators a foundation that is modifiable for their educational needs. As with other assessment tools, validation research will be useful to inform revisions and to provide information on milestones use.^19^ Research may explore metrics such as interrater reliability among faculty or correlation of these milestones with other metrics of successful clinical training. Implementation followed by rigorous assessment and refinement can allow the milestones to improve movement disorders training and subsequent clinical care.

## Author Roles

J.R.: Writing of the first draft; background research; execution of milestone development; review and critique of the manuscript.

S.M.S.: Organization of milestone development; execution of milestone development; review and critique of manuscript.

S.C.: Organization of milestone development; execution of milestone development; review and critique of manuscript.

J.C.: Execution of milestone development; review and critique of manuscript.

C.H.: Execution of milestone development; review and critique of manuscript.

N.O.: Execution of milestone development; review and critique of manuscript.

A.S.: Execution of milestone development; review and critique of manuscript.

E.M.: Execution of milestone development; review and critique of manuscript.

C.S.: Execution of milestone development; review and critique of manuscript.

S.P.: Execution of milestone development; review and critique of manuscript.

P.K.P.: Execution of milestone development; review and critique of manuscript.

L.Y.: Conception of milestone development; organization of milestone development; execution of milestone development; review and critique of manuscript.

## Financial Disclosures of All Authors for the Past 12 Months

J.R. is the deputy section editor for podcasts for the journal *Neurology*. N.O. receives a research grant from The Michael J. Fox Foundation; and honorarium from International Parkinson and Movement Disorders Society. E.M. receives honoraria from Abbott, Medtronic, and Kyowa for consulting services and honorarium from Springer as associate editor of *NPJ Parkinson's Disease*. L.Y. receives grants from Biogen, Sanofi, Alzheimer's Disease Research Center, Pacific Udall Center, and Eli Lily. S.C. is a consultant for Abbott Neuromodulation and consultant medical director for Skypass Foundation. J.C. receives research grant funding and honoraria from Medtronic. All other authors have nothing to declare.

## Financial Disclosure/Conflict of Interest

The authors have no financial disclosures relevant to this submitted manuscript.

## Funding Sources

This project and manuscript did not receive any outside funding.

## Supporting information


**Supplement S1** October 2020 AAN

**Supplement S2**: Movement Disorder Milestones

## Data Availability

No data used for this manuscript. Expert opinion.
