# Supplementary material for: Viewpoint on Milestones for Fellowship Training in Movement Disorders
Source: Mov Disord. 2022 Jul 11;37(8):1605–9. doi: 10.1002/mds.29146 (PMC9543200; doi:10.1002/mds.29146)
Supplement: Supplementary file 1 — Supplement S1 October 2020 AAN [file MDS-37-1605-s001.docx]

**RECOMMENDED CORE CURRICULUM FOR**

**MOVEMENT DISORDERS FELLOWSHIP TRAINING**

**Introduction**

Specialists in Movement Disorders possess higher level knowledge and expertise in the science, clinical evaluation and management of movement disorders. These are conditions that affect the basal ganglia and their direct or indirect connections to the cortex, subcortex, brainstem, cerebellum and spinal cord. This field encompasses the pathophysiology, diagnosis, and treatment of these disorders at a level that is significantly beyond the training and knowledge of a general neurologist.

This document seeks to outline the suggested scope of a comprehensive fellowship curriculum in Movement Disorders. Although individual programs have diverse approaches and strengths, it is proposed that each program strive to provide the most comprehensive exposure and education possible within its unique offerings and environment.

**I. Definition of Movement Disorders**

The specialty of Movement Disorders neurology focuses on neurological disorders that share the common clinical feature of involuntary or abnormal movements of either hypo- or hyperkinetic character. Movement Disorders are classified first phenomenologically and then etiologically. The alterations of movement generally occur in the absence of weakness, and therefore these disorders were originally distinguished by the term “extrapyramidal”, although this term has been largely dropped from current nosography. In terms of phenomenology, hypokinetic Movement Disorders include Parkinson’s disease, several other conditions with parkinsonian features, and rare disorders like Stiff-person syndrome. The large number of hyperkinetic Movement Disorders are divided into several categories including tremor, chorea, dystonia, ataxia, tics, myoclonus and stereotypies.

Movement Disorders are further classified by their causative processes, and include neurodegenerative, genetic, infectious, immune, metabolic, nutritional, toxicological, and vascular etiologies. As such, Movement Disorders may be considered primary when they occur as an isolated neurological syndrome or secondary when they occur as part of a larger process of known cause. In particular, because some Movement Disorders are drug-induced and iatrogenic in etiology, experts must be particularly aware of the agents that are associated with their induction.

Movement Disorders neurology bridges basic science with clinical care and practice. It encompasses several aspects of basic science, including neuroepidemiology, genetics, molecular biology, neurochemistry, neuropharmacology and neurophysiology. In addition, because most Movement Disorders are complex and often chronic conditions, expert clinical care involves familiarity with the recognition and management of motor symptoms as well as cognitive and behavioral comorbidities. One must develop skill in directing appropriate care and incorporating pharmacologic, neuro-rehabilitative, and surgical therapies as appropriate. Further, clinicians must develop skills in communicating and counseling patients and families navigating their experience in movement disorders.

With expanding applications of procedural and neurosurgical interventions for the treatment of Parkinson’s disease, dystonia and various forms of tremor, Movement Disorders specialists must be skilled in identifying candidates for botulinum toxin therapy and when appropriate, for surgical therapies, and conduct appropriate pre- and post-operative neurological management. This includes counseling patients on risks and benefits of procedures, and being prepared to recognize and manage complications should they arise. Because some Movement Disorders have typical imaging patterns, especially various secondary Movement Disorders, neuroimaging expertise also closely integrates in the daily practice of Movement Disorders as a neurological specialty.

**II. Core Content of Movement Disorders Fellowships**

a. Goals and Objectives

The major goal of a Movement Disorders curriculum is to develop a specialized teaching program in the areas of patient care, clinical and/or basic science research, teaching, and education. The field of Movement Disorders, with its numerous conditions, lends itself to further sub-specialization by phenomenological entities. However, given the broad nature of the field, this document primarily addresses a comprehensive general curriculum for training in Movement Disorders, considered as a single sub-specialty, and leaves open the possibility of expansion of the curriculum by programs or individuals with more selective and in-depth interests. The curriculum is oriented at the level of post-residency fellows, but can be adapted for other types of training.

The major objective of a Movement Disorders curriculum is to delineate training that will cultivate expertise in the recognition, diagnosis, treatment, management, and rehabilitation of inpatients and outpatients with Movement Disorders. Exposure to the following components of neurology, neurobiology and allied disciplines are recommended as they apply specifically to Movement Disorders:

• Clinical management of adult and pediatric movement disorders (Programs may not include regular pediatric care, but basic education in conditions that emerge in childhood is encouraged.)

• Emergency management of acute and chronic movement disorders.

• Psychiatry in movement disorders, including movement disorders emerging in primary psychiatric conditions, such as drug-induced and psychogenic movement disorders, and initial management of psychiatric symptoms (mood and behavior) in primary movement disorders.

• Behavioral neurology or neuropsychology, for the diagnosis and management of cognitive and language disturbances that associate with some movement disorders

• Neurosurgical therapies for movement disorders

• Neurorehabilitation for acute and chronic movement disorders

• Neuroimaging (MR, CT, DaT, PET) patterns in movement disorders

• Neuroepidemiology: populational patterns and epidemiological issues

• Broad concepts of neuropharmacology and neurochemistry, particularly with regard to clinically relevant neurotransmitters

• Neuropathology/Cellular biology as it relates to neurodegeneration, apoptosis, and underlying processes important to movement disorders

• Neurological Education: teaching experience for physicians, health professionals, patients and the public in movement disorders including counseling patients on a day-to-day basis

• Exposure to telehealth delivery

b. Prerequisites for Training

Fellowships in Movement Disorders are post-residency positions that are reserved for licensed physicians who have successfully completed neurology residency. Foreign medical graduates may receive a waiver of this prerequisite based on approval by the fellowship Program Director. Other specialties such as pediatrics, psychiatry and others may qualify on an individual basis.

c. Duration of Training

The minimal period of training will be one year. Depending on the specific goals of individual programs, training periods may be longer. Commonly, in order to incorporate adequate research exposure and experience, additional time is added.

d. Training Program

*1. Institutional Requirement*

The Movement Disorders fellowship must be conducted under the auspices of an approved neurology residency training program within an accredited medical school, a hospital affiliated with a medical school, or a non-medical school environment that meets all other requirements. The fellowship must have the support of the Chairperson of the respective Department of Neurology (or equivalent) and appropriate key personnel of the institution. The training institution should ideally have inpatient services, outpatient services, a critical care unit, neuroimaging facilities, neurorehabilitation services, and clinical or basic research laboratories applicable to Movement Disorders. To qualify as a site for Movement Disorders fellowship training, active patient care, research and educational activities must all be present. Institutional clinical faculty will ideally include neurologists, neurosurgeons, neurorehabilitation specialists, neuropsychologists and/or behavioral neurologists and psychiatrists with interaction as a Movement Disorders team. Scientists will vary in their expertise and composition, but sufficient exposure should be available for interaction with the fellow. In the event that the core faculty or institutional components are partly missing from the sponsoring institution itself, the fellowship director may arrange for critical training to occur at another institution with official arrangements documented. This may also include incorporating web-based training experiences to enhance learning.

*2. Training Program Faculty*

• Program Director (PrD). The PrD must be a board-certified neurologist and possess sufficient clinical, research, educational and administrative ability to direct the fellowship program. Ideally, the PrD himself/herself will be a recognized leader in the field of Movement Disorders neurology. The PrD must be a full-time faculty member in the sponsoring institution and should be available on a regular basis to interact directly with and supervise the fellow’s progress.

• Primary Faculty. Primary training program faculty will be neurologists who are board-certified or board eligible and spend the majority of their neurological commitment in the study and treatment of Movement Disorders and related issues. They must have sufficient protected time, administrative support and commitment to mentor fellows. They must have access to sufficient patients and the ability to teach fellows. At each fellowship program there should be at least one, and preferably more, primary clinical faculty members who, along with the PrD, will provide a diverse and in-depth fellowship experience.

• Support Faculty. Other institutional faculty may include clinical specialists in neurosurgery, neuroimaging, neurorehabilitation, neurobehavior and neuropsychology, neuroepidemiology, genetics, critical care medicine, psychiatry and social work. Each clinical faculty member training a Movement Disorders fellow must be board certified or appropriately certified in the respective field of expertise. Research faculty may relate to the above fields and also include specialists in molecular biology, neurotoxicology, neuropharmacology, neurochemistry, neurophysiology and related areas. Such support faculty may or may not be members of the core institution faculty, but must have a commitment to training fellows to the extent required by the PrD. At least one, and preferably more, basic science support faculty should be directly involved in the Movement Disorders fellowship mentorship.

*3. Method of Teaching*

Fellows will be trained clinically through direct patient contact in outpatient Movement Disorders clinics and potentially inpatient consultations. They will be supervised by trained faculty. The Movement Disorders clinic will provide opportunities to evaluate a wide variety of movement disorders including common ones such as Parkinson's disease, cervical dystonia, essential tremor syndrome, and more rare disorders such as atypical parkinsonian disorders, Huntington disease, Gilles de la Tourette's syndrome, ataxias, etc.

They will also be involved in teaching conferences, symposia, video-rounds, seminars, and lectures that focus on Movement Disorders. They will learn through reading assignments, and journal club discussions are encouraged for teaching critical review of the literature. The focal point of the general Movement Disorders training will be clinical experiences with one-on-one mentorship by the PrD and primary faculty. Special areas of subspecialty training will be arranged by work with the support faculty.

e. Timetable for training

Movement Disorders fellowships will last a minimum of one year and will be longer for individual programs. In the one-year fellowship, at least nine months must be involved with full-time direct patient care (which may include outpatient or inpatient).

f. Methods of Evaluation of the Trainee

The PrD or designated primary faculty member will be responsible for meeting with the fellow at least every six months, and ideally every three months to provide regular feedback on performance and to advise the fellow about strengths and weaknesses. The information on performance will be obtained by contact with the faculty and staff involved with the fellow over the past months since the prior evaluation. A final written evaluation will be provided by the PrD at the end of the fellowship. This evaluation will verify that the fellow has demonstrated sufficient professional ability to practice competently and independently in the field of Movement Disorders neurology. This final document will be part of the fellow’s permanent record retained by the institution.

g. Methods of Evaluating the Fellowship Training Process

In the absence of a formal Movement Disorders board certification mechanism or oversight group whose purpose is to monitor and evaluate Movement Disorders fellowship programs, a self-evaluation program must be instituted within each program. This process may take several forms:

• Yearly retreat for faculty to critique the perceived strengths and weaknesses of training and to solicit suggestions for upgrading or improving the program

• Annual feedback from fellows who are in the program as well as those who have completed the program and are now in their careers. This process can be open-ended with a letter from each fellow, or be documented in a standard form.

• Outside reviewers may be invited to visit the program and critique it with a written list of suggestions for improvements

• Other quality assurance methods, including review of academic or practice positions secured by graduates of the fellowship program, number of publications, research grants obtained, or practice success in the first years after fellowship training

• Documenting continuing education throughout the fellowship (see below), with the fellow being responsible for documenting the educational activity and delivering those documents to the PD.

• Faculty should be engaged in continuing education and academic work.

Documents on this evaluation process should be kept as part of the institutional file on the Movement Disorders fellowship.

h. Mechanisms for feedback

Fellows will complete evaluations of the faculty and curriculum at least every six months, and ideally every three months during the fellowship. In addition, the PD or designated primary faculty member will be available in between these meetings on a weekly basis to discuss any concerns by the fellow or other staff members regarding the fellowship.

i. Methods for upgrading knowledge

The faculty and fellows within the program will participate in continuing education in order to expand their knowledge base and remain up-to-date in their expertise of Movement Disorders.

Activities to accomplish this goal may include:

• Active participation in clinical discussion, rounds and conferences that stimulate discussion and scholarship

• Participation in journal clubs and research conferences

• Review of AAN guidelines relevant to Movement Disorders

• Active participation in professional and scientific societies at the local, regional, national or international level particularly in the form of attendance to meetings and publication of materials in their respective journals.

• Participation in clinical or basic science research programs in Movement Disorders or its related neurobiology.

• Participation in online learning. The International Parkinson Disease and Movement Disorders Society offers free membership to fellows, giving access to online learning modules, evidence-based reviews, MDS journals, and the video library.

• Participation in continuing medical education (see below)

j. Continuing Medical Education needed

At least 20 hours of Category 1 of continuing education in Movement Disorders must be completed annually by the Movement Disorders fellow. The appropriate documents verifying these credits should be given to the PD.

k. Curriculum necessities

*I. Anatomy, neurochemistry and neurophysiology of the basal ganglia*

• Basal ganglia-thalamo-cortical circuits

• Basal ganglia interactions with the brain stem and cerebellum

• Neurotransmitter chemistry and pharmacology: dopamine, acetylcholine, gamma-amino butyric acid (GABA), glutamate, norepinephrine, serotonin

• Electrophysiology of basal ganglia function

• Anatomical, neurochemical and physiological hypotheses related to hypokinesia and hyperkinesia

*II. Clinical Evaluation of Movement Disorders*

• Skills to augment a complete general neurological examination with the aspects of the examination relevant to movement disorders

• Skills to recognize and document patterns of clinical findings in movement disorders focusing on movements at rest and during task performance, and examination of muscle tone, dexterity, posture, and gait with regard to normal and abnormal movements

• Skills to recognize different phenomenologies of movement disorders (tremor, chorea, dystonia, myoclonus, tics, gait disturbances, etc.)

• Skills to assess and differentiate tremor sub-types

• Skills to identify and manage functional movement disorders

• Skills for screening of cognition and mood disorders presenting comorbidly with movement disorders

• Skills to elicit history regarding symptoms associated with movement disorders which can aid diagnosis (such as anosmia, REM behavior disorder, family history, etc.)

• Familiarity with and ability to apply clinical rating scales for movement disorders

• Specifically, expertise in the definition and recognition of the following neurological phenomena is considered essential:

Hypokinesia (akinesia and bradykinesia)

Hyperkinesia

Tremor

Chorea

Choreoathetosis

Ballism

Tics

Stereotypies

Akathisia

Myoclonus

Hemifacial Spasm

Dystonia

Parkinsonism

Restless legs syndrome

REM-sleep behavior disorder

Ataxia

*III. Diagnosis, treatment, and scientific understanding of neurological disorders that are considered within the specialty of Movement Disorders neurology*

For each of the Movement Disorders listed below, the following areas of training should be covered:

Pathogenesis and Pathophysiology, including molecular biology and genetic issues

Epidemiology and risk factors

Clinical features

Co-morbid conditions and non-motor symptoms

Diagnostic evaluation: neuroimaging, laboratory studies

Differential diagnosis

Treatment: pharmacological, surgical, rehabilitative

For pharmacologic options, potential side effects and interactions

Prognosis and natural history

Current areas of research

Hypokinetic Movement Disorders and Syndromes

• Parkinson’s Disease

• Atypical parkinsonian disorders: Multiple system atrophy, Progressive supranuclear palsy, Cortico-basal degeneration , Dementia with Lewy Bodies, Normal pressure hydrocephalus, Drug-induced parkinsonism, Vascular parkinsonism

• Stiff-person syndrome and its variants

• Primary progressive freezing of gait

Hyperkinetic Movement Disorders

• Huntington’s disease

• Other prototypical forms of chorea: Sydenham’s chorea, chorea gravidarum, Lupus chorea, tardive dyskinesia, neuro-acanthocytosis, Huntington disease-like syndrome

• Isolated dystonia: Genetic (e.g. DYT-1), Acquired (e.g. drug-induced), Idiopathic

• Combined forms of dystonia: Genetic (e.g. NBIA), Acquired (e.g. cerebral palsy)

• Gilles de la Tourette syndrome

• Other primary tic disorders

• Secondary tic disorders

• Stereotypies seen in primary psychiatric illnesses

• Stereotypies seen in neurological conditions

• Painful legs/moving toes

• Action and non-rest tremors: Essential or familial tremor, physiological tremor, drug-induced tremors, tremors of metabolic and medical illnesses, rubral tremor, orthostatic tremor, tremors in cerebellar disorders

• Wilson’s disease

• Hemifacial spasm

• Essential Myoclonus

• Secondary myoclonus

• Startle syndromes

• Gait disorders

• Spinal cerebellar ataxias and other forms of ataxias

• Paroxysmal dyskinesias

• Restless leg syndrome

• Akathisia

•Drug and toxin induced Movement Disorders

•Autoimmune and paraneoplastic movement disorders

Symptoms or syndromes associated with movement disorders

• Mild cognitive impairment

• Dementia

• Aphasia

• Depression

• Anxiety

• Impulse control disorder

• Psychosis

• Mania or euphoria

• Emotional lability (pseudobulbar affect)

• Orthostatic hypotension

• Anosmia

• Constipation

• Urinary difficulties (urgency, nocturia, incontinence)

• Sleep disturbances (REM-behavior disorder, restless legs, hypersomnolence, interrupted sleep)

*IV. Special Procedures*

Botulinum toxin: Understanding of therapeutic mechanisms, available toxins, indications, injection techniques and basic approach to forming an individualized treatment plan are critical. This includes formulating an appropriate plan for muscle targeting and dosing taking into consideration age, prior exposure, and symptom severity. It is recommended that treatment of blepharospasm, hemifacial spasm, cervical and limb dystonia, and sialorrhea be covered. Depending on local practice, additional applications such as jaw dystonia and spasticity may be covered. Familiarity with guidance techniques which may include EMG or ultrasound are recommended.

Deep brain stimulation (DBS) programming: interrogating a device, determining thresholds, and programming for therapeutic benefit are core skills to be learned in fellowship. Specific targets for different indications should be covered, including potential pitfalls of misplaced leads and stimulation-induced side effects. Furthermore, screening for, recognizing, and managing other complications of therapy including infection, lead fractures, and detriment to gait, speech, or cognition is essential. Intraoperative testing and microelectrode recording are additional valuable skills that may be included in fellowship training.

Some programs may be able to incorporate education regarding building a surgical program. This may require additional time to achieve, and is considered optional but potentially valuable.

Infusion therapies: For example, continuous duodenal infusion of levodopa; recognizing appropriate candidates, knowing how to direct a patient that wishes to utilize the therapy, approach to dosing and managing treatment and complications (recommended but not required). Where available, apomorphine infusion and evolving subcutaneous infusion therapies may be covered.

Kinematics are increasingly being applied to characterize movement disorders, including the use of surface EMG and wearable devices. At centers where these techniques are utilized, exposure can be a valuable experience to fellows.

*V. Additional Recommendations*

Research: Exposure to clinical trials and methods is strongly recommended. This ought to include understanding of phases of trials, interplay between sponsors and investigators, and participating in recruiting for trials if appropriate.

Scholarly Activity: Conducting or participating in research and writing or presenting scholarly activity is strongly encouraged. A general goal of preparing one book chapter or review, one data article (case series, retrospective chart review, or prospective study), and submitting at least one abstract to a regional, national, or international meeting is recommended. Further, it is recommended that if possible, the fellow practice reviewing an article along with a faculty member. This can be a valuable exercise in critical review of literature and in preparation for being a reviewer in the future.

**APPENDIX I: SUGGESTED CORE READING AS PART OF MOVEMENT DISORDERS CURRICULUM**

*These sections should be updated regularly to assist fellowship programs with a general reading list that covers the major areas of Movement Disorders Fellowship training. LAST UPDATED October 2020.*

**All practice guidelines from the American Academy of Neurology in the Movement Disorder section are recommended. These span Parkinson disease, essential tremor, Tourette syndrome, use of botulinum neurotoxin and more. Currently the following link can be used to access guidelines in movement disorders, found on the AAN website:** <https://www.aan.com/Guidelines/Home/ByTopic?topicId=17>.

**Furthermore, the International Parkinson and Movement Disorder Society has a series of evidence-based publications. These include Treatments for Motor and Non-Motor Symptoms of Parkinson’s Disease, Restless Legs, Essential Tremor, and more. On the society website, there is an option for “MDS Education ROADMAP,” where the learner may select level of expertise and be offered videos, papers, and webcasts of core educational value. The beginner section is a great starting point, and the intermediate section has links to the evidence-based publications which are great core readings. Note, membership is required, but FREE to fellows!**

***Recommended reading is grouped by subject areas. Note that the most strongly recommended core reading is designated with **.***

**Basal ganglia**

Alexander GE, DeLong MR, Strick PL. Parallel organization of functionally segregated circuits linking basal ganglia and cortex. Annual Review of Neuroscience 1986;9:357.

Aron AR, Herz DM, Brown P, Forstmann BU, Zaghloul K. Frontosubthalamic Circuits for Control of Action and Cognition. J Neurosci. 2016 Nov 9;36(45):11489-11495.

**DeLong MR, Wichmann T. Basal Ganglia Circuits as Targets for Neuromodulation in Parkinson Disease. JAMA Neurol. 2015 Nov;72(11):1354-60.

Levy R, Hazrati LN, Herrero MT: Reevaluation of the functional anatomy of the basal ganglia in normal and parkinsonian states. Neuroscience 1997; 76:335-343.

**Obeso JA, Rodriguez-Oroz MC, Stamelou M, Bhatia KP, Burn DJ. The expanding universe of disorders of the basal ganglia. Lancet. 2014 Aug 9;384(9942):523-31

**Obeso JA, Rodriguez-Oroz MC, Benitez-Temino B, et al. Functional organization of the basal ganglia: therapeutic implications for Parkinson's disease. Mov disorders. 2008;23 Suppl 3:S548-559.

Parent A, Hazrati LN: Functional anatomy of the basal ganglia. The cortico-basal ganglia-thalamo-cortical loop. Brain Research 1995; 20:91-127.Parkinson Study Group: Effects of tocopherol deprenyl on the progression of disability in early Parkinson's disease. N Eng J Med, 1993;328:176-183. (DATATOP study)

Seeman P, Van Tol HHM: Dopamine receptor pharmacology. Trends Pharmocol Sci 1994; 15:264-270.

Wong JK, Middlebrooks EH, Grewal SS, Almeida L, Hess CW, Okun MS. A Comprehensive Review of Brain Connectomics and Imaging to Improve Deep Brain Stimulation Outcomes. Mov Disord. 2020;35(5):741-751. doi:10.1002/mds.28045

**Parkinsonism**

**Parkinsonism – Criteria/Definition**

***Parkinson disease-***

**Berg D, Postuma RB, Adler CH,et al. **MDS research criteria for prodromal Parkinson's disease.** Mov Disord. 2015 Oct;30(12):1600-11.

Litvan I, Goldman JG, Tröster AI, e al. **Diagnostic criteria for mild cognitive impairment in Parkinson's disease: Movement Disorders Society Task Force guidelines.** Mov Disord. 2012 Mar;27(3):349-56

**Postuma RB, Berg D, Adler CH, et al., **The new definition and diagnostic criteria of Parkinson's disease.** Lancet Neurol. 2016 May;15(6):546-8.

***Dementia with Lewy bodies-***

**McKeith IG, Ferman TJ, Thomas AJ, Blanc F, Boeve BF, Fujishiro H, Kantarci K, Muscio C, O'Brien JT, Postuma RB, Aarsland D, Ballard C, Bonanni L, Donaghy P, Emre M, Galvin JE, Galasko D, Goldman JG, Gomperts SN, Honig LS, Ikeda M, Leverenz JB, Lewis SJG, Marder KS, Masellis M, Salmon DP, Taylor JP, Tsuang DW, Walker Z, Tiraboschi P; prodromal DLB Diagnostic Study Group. **Research criteria for the diagnosis of prodromal dementia with Lewy bodies.** Neurology. 2020 Apr 28;94(17):743-755. doi: 10.1212/WNL.0000000000009323. Epub 2020 Apr 2. PMID: 32241955; PMCID: PMC7274845.

**McKeith IG, Boeve BF, Dickson DW, Halliday G, Taylor JP, Weintraub D, Aarsland D, Galvin J, Attems J, Ballard CG, Bayston A, Beach TG, Blanc F, Bohnen N, Bonanni L, Bras J, Brundin P, Burn D, Chen-Plotkin A, Duda JE, El-Agnaf O, Feldman H, Ferman TJ, Ffytche D, Fujishiro H, Galasko D, Goldman JG, Gomperts SN, Graff-Radford NR, Honig LS, Iranzo A, Kantarci K, Kaufer D, Kukull W, Lee VMY, Leverenz JB, Lewis S, Lippa C, Lunde A, Masellis M, Masliah E, McLean P, Mollenhauer B, Montine TJ, Moreno E, Mori E, Murray M, O'Brien JT, Orimo S, Postuma RB, Ramaswamy S, Ross OA, Salmon DP, Singleton A, Taylor A, Thomas A, Tiraboschi P, Toledo JB, Trojanowski JQ, Tsuang D, Walker Z, Yamada M, Kosaka K. **Diagnosis and management of dementia with Lewy bodies: Fourth consensus report of the DLB Consortium.** Neurology. 2017 Jul 4;89(1):88-100. doi: 10.1212/WNL.0000000000004058. Epub 2017 Jun 7. PMID: 28592453; PMCID: PMC5496518.

***Corticobasal degeneration-***

**Armstrong MJ, et al. **Criteria for the diagnosis of corticobasal degeneration.** Neurology 2013;80:496-503.

Compston A. From the archives. Corticobasal degeneration. By WRG Gibb, PJ Luther and CD Marsden. Brain 1989: 112; 1171-1192 with Corticobasal degeneration. A clinical study of 36 cases. By JO Rinne, MS Lee, PD Thompson and CD Marsden. Brain 1994: 117; 1183-1196. Brain. 2010 Jul;133(Pt 7):1860-2. doi: 10.1093/brain/awq173. PMID: 20597145.

Litvan I, Agid Y, Goetz C, et al: **Accuracy of the clinical diagnosis of corticobasal degeneration: A clinicopathologic study.** Neurology 1997;48:119-125.

***Multiple system atrophy-***

**Fanciulli A, Wenning GK. Multiple-system atrophy. The New England journal of medicine. 2015;372(3):249-63.

**Gilman, S., G. K. Wenning, P. A. Low, et al). "Second consensus statement on the diagnosis of multiple system atrophy." Neurology 2008; 71(9): 670-676. (Also see critique by Stankovic et.al., below).

Kollensperger M, et al. Red flags for multiple system atrophy. Mov Disorders 2008; 23(8):1093-1099.

Stankovic I, Quinn N, Vignatelli L, Antonini A, Berg D, Coon E, Cortelli P, Fanciulli A, Ferreira JJ, Freeman R, Halliday G, Höglinger GU, Iodice V, Kaufmann H, Klockgether T, Kostic V, Krismer F, Lang A, Levin J, Low P, Mathias C, Meissner WG, Kaufmann LN, Palma JA, Panicker JN, Pellecchia MT, Sakakibara R, Schmahmann J, Scholz SW, Singer W, Stamelou M, Tolosa E, Tsuji S, Seppi K, Poewe W, Wenning GK; Movement Disorder Society Multiple System Atrophy Study Group. A critique of the second consensus criteria for multiple system atrophy. Mov Disord. 2019 Jul;34(7):975-984. doi: 10.1002/mds.27701. Epub 2019 Apr 29. PMID: 31034671; PMCID: PMC6737532.

***Progressive supranuclear palsy-***

**Coughlin DG, Litvan I. Progressive supranuclear palsy: Advances in diagnosis and management. Parkinsonism Relat Disord. 2020 Apr;73:105-116. doi: 10.1016/j.parkreldis.2020.04.014. Epub 2020 May 25. PMID: 32487421; PMCID: PMC7462164.

**Höglinger GU, Respondek G, Stamelou M, et al. Clinical diagnosis of progressive supranuclear palsy: the Movement Disorder Society criteria. *Mov Disord* 2017;**32**(6):853‐864.

Litvan I, Agid Y, Calne D, et al. Clinical research criteria for the diagnosis of progressive supranuclear palsy (Steele-Richardson-Olszewski syndrome): Report of the NINDS-SPSP International Workshop. Neurology 1996;47:1-9.Williams DR, Lees AJ. Progressive supranuclear palsy: clinicopathological concepts and diagnostic challenges. Lancet Neurol 2009;8:270-279.

**Parkinsonism – Pharmacological Treatment**

Cilia R, Akpalu A, Sarfo FS, et al. The modern pre-levodopa era of Parkinson's disease: insights into motor complications from sub-Saharan Africa. *Brain*. 2014;137(Pt 10):2731-2742. doi:10.1093/brain/awu195

**Connolly BS, Lang AE. Pharmacological treatment of Parkinson disease: a review. JAMA. 2014;311(16):1670-83.

Fahn S; Parkinson Study Group. Does levodopa slow or hasten the rate of progression of Parkinson’s disease? J Neurol. 2005 Oct;252 Suppl 4:IV37-IV42. (ELLDOPA study)

**Jankovic J, Tan EK. Parkinson’s disease: Etiopathogenesis and treatment. J Neurol Neurosurg Psychiatry. 2020;91(8):795-808. Doi:10.1136/jnnp-2019-322338 Kalia LV, Lang AE. Parkinson’s disease. Lancet 2015;386:896-912.

Olanow CW, Rascol O, Hauser R, Feigin PD, Jankovic J, Lang A, Langston W, Melamed E, Poewe W, Stocchi F, Tolosa E. A double-blind, delayed-start trial of rasagiline in Parkinson's disease. The New England journal of medicine. 2009;361(13):1268-78. (ADAGIO study)

Parkinson Study Group. Pramipexole vs levodopa as initial treatment for Parkinson disease: A randomized controlled trial. JAMA. 2000 Oct 18;284(15):1931-8. (CALM-PD study)

Parkinson Study Group. A controlled trial of rasagiline in early Parkinson disease: the TEMPO Study. Arch Neurol. 2002 Dec;59(12):1937-43. (TEMPO study)

Sethi KD, O=Brien CF, Hammerstad JP, Adler CH, Davis TL, Taylor RL, Sanchez-Ramos J, Bertoni JM, Hauser RA. Ropinirole for the treatment of early Parkinson disease: a 12-month experience. Ropinirole Study Group. Arch Neurol 1998; 55:1211-1216.

Stocchi F, Rascol O, Kieburtz K, Poewe W, Jankovic J, Tolosa E, Barone P, Lang AE, Olanow CW. Initiating levodopa/carbidopa therapy with and without entacapone in early Parkinson disease: the STRIDE-PD study. Ann Neurol. 2010 Jul;68(1):18-27. doi: 10.1002/ana.22060. Erratum in: Ann Neurol. 2010 Sep;68(3):412-3.

Verschuur CVM, Suwijn SR, Boel JA, Post B, Bloem BR, van Hilten JJ, van Laar T, Tissingh G, Munts AG, Deuschl G, Lang AE, Dijkgraaf MGW, de Haan RJ, de Bie RMA; LEAP Study Group. Randomized Delayed-Start Trial of Levodopa in Parkinson's Disease. N Engl J Med. 2019 Jan 24;380(4):315-324. doi: 10.1056/NEJMoa1809983. PMID: 30673543.

**Parkinsonism – Non Motor Symptoms**

Barone P, Antonini A, Colosimo C, Marconi R, Morgante L, Avarello TP, et al. The PRIAMO study: A multicenter assessment of nonmotor symptoms and their impact on quality of life in Parkinson’s disease. Mov Disord. 2009 Aug 15;24(11):1641-9.

**Fox SH, Katzenschlager R, Lim SY, Barton B, de Bie RMA, Seppi K, Coelho M, Sampaio C; Movement Disorder Society Evidence-Based Medicine Committee. International Parkinson and movement disorder society evidence-based medicine review: Update on treatments for the motor symptoms of Parkinson’s disease. Mov Disord. 2018 Aug;33(8):1248-1266. Doi: 10.1002/mds.27372. Epub 2018 Mar 23. Erratum in: Mov Disord. 2018 Dec;33(12):1992. PMID: 29570866.

Reid WG, Hely MA, Morris JG, Loy C, Halliday GM. Dementia in Parkinson's disease: a 20-year neuropsychological study (Sydney Multicentre Study). J Neurol Neurosurg Psychiatry. 2011 Sep;82(9):1033-7. doi: 10.1136/jnnp.2010.232678. Epub 2011 Feb 18.

**Seppi K, Ray Chaudhuri K, Coelho M, et al. Update on treatments for nonmotor symptoms of Parkinson’s disease—an evidence-based medicine review. Mov Disord. 2019;34(2):180-198. doi:10.1002/mds.27602

Voon V, Sohr M, Lang AE, Potenza MN, Siderowf AD, Whetteckey J, Weintraub D, Wunderlich GR, Stacy M. Impulse control disorders in Parkinson disease: a multicenter case--control study. Ann Neurol. 2011 Jun;69(6):986-96. doi: 10.1002/ana.22356. Epub 2011 Mar 17. (DOMINION)

Zuzuárregui JRP, During EH. Sleep Issues in Parkinson's Disease and Their Management. Neurotherapeutics. 2020 Oct 7. doi: 10.1007/s13311-020-00938-y. Epub ahead of print. PMID: 33029723.

**Parkinsonism – Others**

Ba F, Martin WR. Dopamine transporter imaging as a diagnostic tool for parkinsonism and related disorders in clinical practice. Parkinsonism Relat Disord. 2015 Feb;21(2):87-94.

Fahn S: The freezing phenomenon in parkinsonism. Adv Neurol 1995; 67:53-63.

Kumar KR, Lohmann K, Klein C. Genetics of Parkinson disease and other Movement Disorders. Current opinion in neurology 2012;25:466-474.

Langston JW, Ballard PA, Tetrud JW: Chronic parkinsonism in humans due to a product of meperidine-analog synthesis. Science 1983;219:979-980.

Lopez-Sendon JL, et al. Drug-induced parkinsonism in the elderly. Incidence, management and prevention. Drugs Aging 2012; 29(2): 105-118.

Polymeropoulos MH, Lavedant C, Leroy E, et al: Mutation in the alpha-synuclein gene identified in families with Parkinson's disease. Science 1997;276:2045-2057.

**Surigcal Management of Parkinson’s disease**

**Deuschl G, Schade-Brittinger C, Krack P, et al; German Parkinson Study Group, Neurostimulation Section. A randomized trial of deep-brain stimulation for Parkinson’s disease. N Engl J Med. 2006 Aug 31;355(9):896-908.

**Follett KA, Weaver FM, Stern M, et al; CSP 468 Study Group. Pallidal versus subthalamic deep-brain stimulation for Parkinson’s disease. N Engl J Med. 2010 Jun 3;362(22):2077-91.

Herrington TM, Cheng JJ, Eskandar EN. Mechanisms of deep brain stimulation. J Neurophysiol. 2016 Jan 1;115(1):19-38.

Krack P, Batir A, Van Blercom N, et al. Five-year follow-up of bilateral stimulation of the subthalamic nucleus in advanced Parkinson’s disease. N Engl J Med. 2003 Nov 13;349(20):1925-34.

Lang AE, Lozano A, Montgomery E, et al: posteroventral medial pallidotomy in advanced Parkinson’s disease. N Engl J Med 1997;337:1036-1042.

Okun MS. Deep-brain stimulation for Parkinson's disease. The New England journal of medicine 2012;367:1529-1538.

Schuepbach WM, Rau J, Knudsen K, et al.; EARLYSTIM Study Group. Neurostimulation for Parkinson's disease with early motor complications. N Engl J Med. 2013 Feb 14;368(7):610-22.

**Tremor**

**Ferreira JJ, Mestre TA, Lyons KE, Benito-León J, Tan EK, Abbruzzese G, Hallett M, Haubenberger D, Elble R, Deuschl G; MDS Task Force on Tremor and the MDS Evidence Based Medicine Committee. MDS evidence-based review of treatments for essential tremor. Mov Disord. 2019 Jul;34(7):950-958. doi: 10.1002/mds.27700. Epub 2019 May 2. PMID: 31046186.

**Giordano M, Caccavella VM, Zaed I, Foglia Manzillo L, Montano N, Olivi A, Polli FM. Comparison between deep brain stimulation and magnetic resonance-guided focused ultrasound in the treatment of essential tremor: a systematic review and pooled analysis of functional outcomes. J Neurol Neurosurg Psychiatry. 2020 Oct 14:jnnp-2020-323216. doi: 10.1136/jnnp-2020-323216. Epub ahead of print. PMID: 33055140.

Louis, E. Continuum: Lifelong Learning in Neurology. 2019;25(4):959-975.

Wardt JV, van der Stouwe AMM, Dirkx M, Elting JWJ, Post B, Tijssen MA, Helmich RC. Systematic clinical approach for diagnosing upper limb tremor. J Neurol Neurosurg Psychiatry. 2020 Aug;91(8):822-830. doi: 10.1136/jnnp-2019-322676. Epub 2020 May 26. PMID: 32457087; PMCID: PMC7402459.

Zesiewicz TA, et al. Evidence-based guideline update: treatment of essential tremor: report of the Quality Standards subcommittee of the American Academy of Neurology. Neurology 2011; 77(19):1752-5.

**Chorea**

A Physician’s Guide to the Management of Huntington’s Disease. Available for free download from [www.hdsa.org](file:///C:\Users\irini\Downloads\www.hdsa.org).

Baizabal-Carvallo JF, Cardoso F.J Neural Transm (Vienna). Chorea in children: etiology, diagnostic approach and management. 2020 Oct;127(10):1323-1342. doi: 10.1007/s00702-020-02238-3. Epub 2020 Aug 9. PMID: 32776155

Ha AD, Fung VS. Huntington's disease. Current opinion in neurology 2012;25:491-498.

**Hermann A, Walker RH. Diagnosis and treatment of chorea syndromes. Curr Neurol Neurosci Rep. 2015;15(2):514. doi: 10.1007/s11910-014-0514-0. PMID: 25620691.

Huntington’s Disease Society of America Free 10 CME Modules. <https://hdsa.org/healthcare-professionals-resources/hd-101-cme-series/>

**Martinez-Ramirez D, Walker RH, Rodríguez-Violante M, Gatto EM. Review of Hereditary and Acquired Rare Choreas. Tremor and Other Hyperkinetic Movements. 2020;10(1):1-24. doi:10.5334/tohm.548

Tabrizi SJ, Flower MD, Ross CA, Wild EJ. Huntington disease: new insights into molecular pathogenesis and therapeutic opportunities. Nat Rev Neurol. 2020 Oct;16(10):529-546. Doi:10.1038/s41582-020-0389-4.

**Termsarasab, Pichet. Chorea. Continuum: Lifelong Learning in Neurology. 2019;25(4):p1001-1035.

Walker RH. The non-Huntington disease choreas: Five new things. Neurol Clin Pract. 2016 Apr;6(2):150-156. doi: 10.1212/CPJ.0000000000000236. PMID: 29377035; PMCID: PMC5720620.

**Tics**

Leckman JF, Peterson BS, Anderson GM, et al: Pathogenesis of Tourette's syndrome. J Child Psychol Psychiatry 1997;38:119-142.

Malaty IA, Akbar U. Updates in medical and surgical therapies for Tourette syndrome. Curr Neurol Neurosci Rep. 2014 Jul;14(7):458.

**Pringsheim T, Holler-Managan Y, Okun MS, Jankovic J, Piacentini J, Cavanna AE, Martino D, Müller-Vahl K, Woods DW, Robinson M, Jarvie E, Roessner V, Oskoui M. Comprehensive systematic review summary: Treatment of tics in people with Tourette syndrome and chronic tic disorders. Neurology. 2019 May 7;92(19):907-915. doi: 10.1212/WNL.0000000000007467. Erratum in: Neurology. 2019 Aug 27;93(9):415. PMID: 31061209; PMCID: PMC6537130.

Roessner V, et al. European clinical guidelines for Tourette syndrome and other tic disorders. Part II: pharmacological treatment. Eur Child Adolesc Psychiatry 2011; 20:173-196.

Singer HS. Tourette's syndrome: from behaviour to biology. The Lancet Neurology 2005;4:149-159.

Xu W, Zhang C, Deeb W, Patel B, Wu Y, Voon V, Okun MS, Sun B. Deep brain stimulation for Tourette's syndrome. Transl Neurodegener. 2020 Jan 13;9:4. doi: 10.1186/s40035-020-0183-7. PMID: 31956406; PMCID: PMC6956485.

**Dystonia**

**Albanese A, Di Giovanni M, Lalli S. Dystonia: diagnosis and management. Eur J Neurol. 2019 Jan;26(1):5-17. doi: 10.1111/ene.13762. Epub 2018 Aug 18. PMID: 30035844.

Albanese A, et al. Phenomenology and classification of dystonia: a consensus update. Mov Disorders 2013; 28(7): 863.

Balint B, Mencacci N, Valente E, Pisani A, Rothwell J, Jankovic J, Vidailhet M, Bhatia K. Dystonia. Nature Reviews Disease Primers. 2018; 4(25). <https://doi-org.laneproxy.stanford.edu/10.1038/s41572-018-0023-6>

Hallett M. Neurophysiology of dystonia: The role of inhibition. Neurobiol Dis. 2011 May;42(2):177-84.

**Kupsch A, Benecke R, Müller J, et al.; Deep-Brain Stimulation for Dystonia Study Group. Pallidal deep-brain stimulation in primary generalized or segmental dystonia. N Engl J Med. 2006 Nov 9;355(19):1978-90.

Ozelius LJ, Hewett JW, Page CE, et al: The early onset torsion dystonia gene (DYT1) encodes an ATP-binding protein. Nature Genet 1997;17:40-48.

**Myoclonus**

**Caviness J. Myoclonus. Continuum: Lifelong Learning in Neurology. 2019;25(4):1055-1080

Lance JW, Adams RD: The syndrome of intention or action myoclonus as a sequel to hypoxic encephalopathy. Brain 1963; 86:111-136.

Lozsadi D. Myoclonus: a pragmatic approach. Practical Neurology 2012;12:215-224.

Mills K, Mari Z. An update and review of the treatment of myoclonus. Current Neurol Neurosci Rep. 2015(1):512

**Ataxia**

Subramony SH. Approach to ataxic diseases. Handbook of clinical neurology. 2012;103:127-34.

Carroll LS, Massey TH, Wardle M, Peall KJ. Dentatorubral-pallidoluysian atrophy: An update. Tremor and Other Hyperkinetic Movements. 2018;8. doi:10.7916/D81N9HST

**Perlman, S. Evaluation and Management of Ataxic Disorders. An Overview for Physician. <https://secureservercdn.net/166.62.112.199/y1x.318.myftpupload.com/wp-content/uploads/2017/07/Evaluation_and_Management_of_Ataxic_Disorders-An_overview_for_Physicians.pdf>

National Ataxia Foundation. <https://ataxia.org/fact-sheets/>

**Miscellaneous**.

Bhatia KP. Paroxysmal dyskinesias. Mov Disord. 2011 May;26(6):1157-65.

**Chirra M, Marsili L, Gallerini S, Keeling EG, Marconi R, Colosimo C. Paraneoplastic movement disorders: phenomenology, diagnosis, and treatment. Eur J Intern Med. 2019 Sep;67:14-23. doi: 10.1016/j.ejim.2019.05.023. Epub 2019 Jun 12. PMID: 31200996.

**Continuum (Minneap Minn). Aug 2019; Vol. 25, No.4.(Movement Disorders). This issue of Continuum is a great resource for an overview of several Movement Disorders.

Dressler D, Thompson PD, Gledhill RF, Marsden CD: The syndrome of painful legs and moving toes. Mov Disord 1994;9:13-21.

**Espay AJ, Aybek S, Carson A, Edwards MJ, Goldstein LH, Hallett M, LaFaver K, LaFrance WC Jr, Lang AE, Nicholson T, Nielsen G, Reuber M, Voon V, Stone J, Morgante F. Current Concepts in Diagnosis and Treatment of Functional Neurological Disorders. JAMA Neurol. 2018 Sep 1;75(9):1132-1141. doi: 10.1001/jamaneurol.2018.1264. PMID: 29868890; PMCID: PMC7293766.

**Factor SA, Burkhard PR, Caroff S, Friedman JH, Marras C, Tinazzi M, Comella CL. Recent developments in drug-induced movement disorders: a mixed picture. Lancet Neurol. 2019 Sep;18(9):880-890. doi: 10.1016/S1474-4422(19)30152-8. Epub 2019 Jul 3. PMID: 31279747

Gandhi SE, Newman EJ, Marshall VL. Emergency presentations of movement disorders. Pract Neurol. 2020 Aug;20(4):practneurol-2019-002277. doi: 10.1136/practneurol-2019-002277. Epub 2020 Apr 16. PMID: 32299832.

Iranzo A, Fernández-Arcos A, Tolosa E, Neurodegenerative disorder risk in idiopathic REM sleep behavior disorder: study in 174 patients. PLoS One. 2014 Feb 26;9(2):

Lorincz MT. Neurologic Wilson’s disease. Ann NY Acad Sci 2010; 1184:173-187.

Matsumoto J, Hallett M: Startle syndromes. In: Marsden CD, Fahn S (eds.): Movement Disorders 3, Oxford, Butterworth-Heinemann, 1994, pp 418-433.

McKeon A, Robinson MT, McEvoy KM, Matsumoto JY, Lennon VA, Ahlskog JE, Pittock SJ. Stiff-man syndrome and variants: clinical course, treatments, and outcomes. Archives of neurology. 2012;69(2):230-8.

Saini AG, Sharma S. Movement Disorders in Inherited Metabolic Diseases in Children. *Ann Indian Acad Neurol*. 2020;23(3):332-337. doi:10.4103/aian.AIAN_612_19

Tanner CM, Goldman SM, Ross GW, Grate SJ. The disease intersection of susceptibility and exposure: chemical exposures and neurodegenerative disease risk. Alzheimers Dement. 2014 Jun;10(3 Suppl):S213-25.

**Book Recommendations:**

Ahlskog, J. Parkinson’s Disease Treatment Guide for Physicians. Oxford University Press; 2009.

Espay AJ, Lang AE. Common Movement Disorders Pitfalls: Case-Based Learning. Cambridge University Press 2012.

Fahn S, Jankovic J, Hallett M. Principles and practice of Movement Disorders. 2nd ed. Edinburgh ; New York: Elsevier/Saunders; 2011.

Jankovic J, Tolosa E. Parkinson's Disease and Movement Disorders Sixth Edition. LWW; 2015.

Marks WJ Jr. Deep Brain Stimulation Management, 2^nd^ edition. Cambridge University Press; 2015.

Marsden’s Book of Movement Disorders. Oxford University Press; 2012. (See comment)

Mehanna R. Deep Brain Stimulation. Nova Science Publishers; 2015.

Perotto AO. Anatomical Guide for the Electromyographer: The Limbs and Trunk 5th Edition. Charles C. Thomas Publisher LTD.; 2011 (for localization in botulinum toxin injections)

Schapira AHV, Lang AET, Fahn H. Movement Disorders 4 (Blue Books of Neurology Series, Vol. 34). Saunders;1^st^ edition; 2010.

Singer HS, Mink JW, Gilbert DL, Janovic J. Movement Disorders in Childhood. Elsevier/Saunders; 2010.

Truong D, Dressler D, Hallett M, Zachary C. Manual of Botulinum Toxin Therapy. Cambridge University Press; 2013.

**Online materials:**

MDS Roadmap

MDS digital educational portfolio

MDS certification in MDS-UPDRS is a valuable experience to ensure appropriate application of the rating scale.
