# Supplementary material for: Viewpoint on Milestones for Fellowship Training in Movement Disorders
Source: Mov Disord. 2022 Jul 11;37(8):1605–9. doi: 10.1002/mds.29146 (PMC9543200; doi:10.1002/mds.29146)
Supplement: Supplementary file 2 — Supplement S2: Movement Disorder Milestones [file MDS-37-1605-s002.pdf]

Glossary of Acronyms:

BG: Basal Ganglia
C/L: Carbidopa/Levodopa
DBS: Deep Brain Stimulation
EMG: Electromyography
ET: Essential Tremor
FMD: Functional Movement Disorder
FUS: Focused Ultrasound
GKRT: Gamma Knife Radiation Therapy
NPH: Normal Pressure Hydrocephalus
OT: Occupational Therapy
PD: Parkinson’s Disease
PT: Physical Therapy/Physiotherapy
QI: Quality Improvement
RLS: Restless Leg Syndrome
SW: Social Work/Social Worker

Patient Care 1: History Taking				
Level 1	Level 2	Level 3	Level 4	Level 5
Obtains a standard neurological and medical history<br><br>Obtains a history of movement disorder symptoms and risk factors	Obtains a complete and relevant history, including hypothesis-driven elements<br><br>Obtains history for key “red flag” symptoms, (e.g. for atypical parkinsonism)<br><br>Obtains history for key non-motor symptoms	Obtains a complete, relevant, and **organized** history<br><br>Screens for safety concerns (swallowing, driving, supervision, falls, etc.)	**Efficiently** obtains a complete, relevant, and organized history<br><br>Probes for patient goals, values, and priorities, including expanded social history	Expertly obtains a complete, relevant, and organized history while incorporating subtle verbal and non-verbal clues

Comments:

Not Yet Completed Level 1

Not Yet Assessable

Patient Care 2: Movement Disorders Examination				
Level 1	Level 2	Level 3	Level 4	Level 5
Performs a basic movement disorders examination in addition to a complete neurologic examination<br><br>Recognizes severely abnormal phenomenology in basic examination<br><br>Demonstrates familiarity with examination-based rating scales	Performs a movement disorders examination accurately<br><br>Recognizes mildly abnormal phenomenology in basic examination<br><br>Incorporates hypothesis-driven general examination maneuvers as needed (e.g. ophthalmologic, neuromuscular, musculoskeletal)<br><br>Uses examination rating scales	Tailors movement disorders examination appropriately to the patient's history and chief complaint<br><br>Uses exam to seek some specialized phenomenology (e.g. optokinetic testing, applause sign)<br><br>Accurately grades examination rating scales	Performs an organized, hypothesis-driven movement disorders examination<br><br>Seeks and recognizes all appropriate phenomenology<br><br>Includes all appropriate neurological and general medical examination maneuvers	Role models the performance of the movement disorders examination to specialty peers

Comments:

Not Yet Completed Level 1

Not Yet Assessable

Patient Care 3: Movement Disorders Formulation				
Level 1	Level 2	Level 3	Level 4	Level 5
Summarizes history, examination findings, and available diagnostic testing	Uses relevant history, examination, and diagnostic testing to develop a broad differential diagnosis<br><br>Incorporates other medical or neurological information as it relates to a movement disorders diagnosis (e.g. hepatic dysfunction in Wilson's)	Uses patient information to develop an appropriately prioritized differential diagnosis<br><br>Recognizes the contribution of other medical or neurological conditions in the presentation (e.g. neuromuscular, orthopedic)	Revises the differential diagnosis based on patient progression or emergence of new information<br><br>Synthesizes information on complex or multifactorial patient presentations	Role models advanced clinical reasoning in complex movement disorder syndromes

Comments:

Not Yet Completed Level 1

Not Yet Assessable

Patient Care 4: Parkinson's Disease (PD)				
Level 1	Level 2	Level 3	Level 4	Level 5
Appropriately includes PD on the differential diagnosis<br><br>Discusses all classes of medications for PD	Describes clinical criteria for PD<br><br>Diagnoses **straight-forward** PD<br><br>Discusses typical PD medication dosing and non-pharmacological options<br><br>Discusses DaTscan imaging patterns	Diagnoses **subtle or mild** PD<br><br>Develops thorough and individualized treatment plan for motor and non-motor symptoms in straight-forward PD patients<br><br>Manages basic motor and non-motor complications of therapy<br><br>Documents consideration of advanced PD therapies (DBS, C/L infusion pump, etc.) and refers for complete pre-DBS evaluation	Manages treatment regimens for refractory or advanced PD patients<br><br>Appropriately chooses between advanced PD therapies including DBS target<br><br>Independently interprets DaTscans	Expertly manages variable types and severities of PD patients, including the use of advanced therapies

Comments:

Not Yet Completed Level 1

Not Yet Assessable

Patient Care 5: Other Parkinsonian Disorders				
Level 1	Level 2	Level 3	Level 4	Level 5
Appropriately includes atypical parkinsonism on the differential diagnosis<br><br>Describes cardinal history, examination, and imaging features of each atypical disorder<br><br>Recognizes possible drug-induced Parkinsonism	Describes diagnostic criteria for atypical disorders (MSA-P, MSA-C, CBS, PSP, DLB, vascular)<br><br>Elicits specific signs for atypical syndromes during physical exam (e.g. OKN for PSP, cortical sensory signs for CBS)<br><br>Diagnoses and initiates treatments for **straight-forward** atypical parkinsonisms<br><br>Demonstrates consideration of secondary Parkinsonism from exposures or metabolic disease (e.g. Normal Pressure Hydrocephalus/NPH, Wilson's, heavy metals)	Diagnoses **subtle** atypical parkinsonian diseases<br><br>Adjusts treatment for motor/non-motor symptoms independently<br><br>Appropriately tests for Parkinsonism from exposures or metabolic diseases	Discusses diagnostic considerations in cases of overlapping syndromes or diagnostic uncertainty<br><br>Manages complicated treatment regimens, including balancing benefit and side effects of medications	Serves as a role model for the diagnosis and management of complex atypical parkinsonism patients

Comments:

Not Yet Completed Level 1

Not Yet Assessable

**Patient Care 6: Tremor**				
Level 1	Level 2	Level 3	Level 4	Level 5
Recognizes tremor phenomenology on examination<br><br>Differentiates tremor subtypes (e.g. rest, postural, kinetic, intention, task-specific)<br><br>Formulates differential diagnosis of primary tremor etiology (e.g. PD, ET, physiologic)<br><br>Discusses first-line medications for tremor treatment	Recognizes **mild** tremor and mixed phenomenology<br><br>Correctly reaches etiological diagnosis (e.g. physiological, essential/ET, rubral)<br><br>Initiates appropriate management of ET and action tremor<br><br>Includes secondary causes of tremor on the differential<br><br>Describes advanced/surgical therapies (DBS/GKRT/FUS)	Uses second-line agents for management of ET and action tremor<br><br>Initiates investigations and management of complex tremor syndromes (e.g. Holmes) including pharmacological and non-pharmacological treatments and chemodenervation<br><br>Documents consideration of advanced/surgical therapies (DBS/GKRT/FUS) and refers for complete pre-DBS evaluation	Manages advanced and refractory tremor using all available treatment options<br><br>Appropriately chooses between advanced therapies	Expertly manages refractory, complex, and mixed phenomenology patients with tremor

Comments:

Not Yet Completed Level 1

Not Yet Assessable

Patient Care 7: Dystonia				
Level 1	Level 2	Level 3	Level 4	Level 5
Appropriately includes dystonia on the differential diagnosis<br><br>Describes and probes for dystonia-specific findings on history and examination (e.g. sensory trick, task-specificity, overflow/mirror movements)<br><br>Discusses basics of dystonia treatment	Diagnoses **severe** dystonia<br><br>Identifies clinical categorization and distribution of dystonia (focal, generalized, segmental, etc.)<br><br>Initiates basic treatment for dystonia including referral for chemodenervation<br><br>Initiates basic workup for dystonia	Recognizes **mild** dystonia and mixed phenomenology<br><br>Includes specific genetic dystonias on the differential diagnosis<br><br>Tailors diagnostic workup to the individual patient including genetic testing<br><br>Formulates and executes individualized treatment plan, and adjusts dystonia treatment based on response	Manages refractory or complex dystonias<br><br>Appropriately documents patient candidacy for DBS	Serves as an expert in the medical and surgical management of complex dystonia patients

Comments:

Not Yet Completed Level 1

Not Yet Assessable

Patient Care 8: Other Hyperkinetic Disorders				
Level 1	Level 2	Level 3	Level 4	Level 5
Appropriately includes hyperkinetic disorders (tics, myoclonus, startle, ballism, hemifacial spasm, Restless Leg/RLS, tardive dyskinesia) on the differential<br><br>Recognizes historical elements to make RLS diagnosis	Distinguishes **straight-forward** hyperkinetic phenomenologies<br><br>Independently initiates diagnostic work up<br><br>Describes and probes for secondary causes (e.g. drugs, toxins, systemic diseases)<br><br>Initiates management of common hyperkinetic disorders<br><br>Discusses rare hyperkinetic syndromes (Startle syndromes, paroxysmal dyskinesias)	Recognizes **mild or mixed** hyperkinetic phenomenologies<br><br>Diagnoses rare syndromes and secondary causes<br><br>Manages refractory common hyperkinetic disorders<br><br>Tailors treatment decisions based on relevant psychiatric and cognitive comorbidities	Manages rare or complex hyperkinetic disorders	Serves as an expert in the management of refractory, rare, or complex hyperkinetic disorders

Comments:

Not Yet Completed Level 1

Not Yet Assessable

**Patient Care 9: Ataxia**				
Level 1	Level 2	Level 3	Level 4	Level 5
Performs appropriate examination maneuvers to assess cerebellar function<br><br>Recognizes **straight-forward** ataxia on examination	Formulates a broad differential diagnosis and initiates diagnostic workup<br><br>Discusses medication options for ataxia treatment	Recognizes **subtle** ataxia on exam<br><br>Appropriately includes genetic, immune-mediated, and rare ataxias on the differential diagnosis<br><br>Tailors diagnostic workup to the individual patient including genetic testing<br><br>Formulates and executes individualized treatment plan	Manages complicated treatment regimens, including balancing benefit and side effects of medications<br><br>Demonstrates sophisticated knowledge of overlap between syndromes and diagnostic uncertainties	Serves as a role model for the management of complex ataxia patients, including anticipatory planning in the case of neurodegenerative ataxias (e.g. care goals, care needs)

Comments:

Not Yet Completed Level 1

Not Yet Assessable

Patient Care 10: Huntington's Disease (HD) and other Choreas				
Level 1	Level 2	Level 3	Level 4	Level 5
Recognizes **severe** chorea on examination<br><br>Describes basics of HD diagnosis including genetics<br><br>Understands indications for genetic testing	Recognizes **mild** chorea on exam<br><br>Recognizes non-motor symptoms associated with HD<br><br>Appropriately refers for HD genetic testing<br><br>Formulates non-HD differential diagnosis of chorea (e.g. other genotypes, systemic disease)	Initiates symptomatic treatment as appropriate<br><br>Appropriately counsels patients/families with HD<br><br>Initiates diagnostic testing for non-HD differential diagnosis of chorea (e.g. other genotypes, systemic disease)<br><br>Discusses the role of multidisciplinary care in HD	Appropriately involves multidisciplinary team<br><br>Independently manages motor and non-motor HD symptoms	Manages multidisciplinary team<br><br>Serves as a role model for the management of complex HD patients, including anticipatory planning (e.g. care goals and care needs) and genetic counseling

Comments:

Not Yet Completed Level 1

Not Yet Assessable

Patient Care 11: Functional Movement Disorders (FMD)				
Level 1	Level 2	Level 3	Level 4	Level 5
Appropriately includes FMD on the differential<br><br>Recognizes red flags that raise suspicion for functional movement disorders	Recognizes **straight-forward** FMD presentations using pertinent examination techniques and relevant historical clues<br><br>Discusses general treatment options for functional disorders	Uses additional techniques to recognize **subtle** functional phenomenology<br><br>Initiates an appropriate treatment plan for functional disorders	Identifies functional phenomenology in the context of other neurologic diseases<br><br>Independently manages functional movement disorders patients with appropriate care team and multidisciplinary services	Role models compassionate, multidisciplinary care of functional movement disorders patients

Comments:

Not Yet Completed Level 1

Not Yet Assessable

Patient Care 12: Therapeutic Chemodenervation				
Level 1	Level 2	Level 3	Level 4	Level 5
Reconstitutes botulinum toxin<br><br>Discusses all botulinum toxin formulations and mechanisms of action<br><br>Discusses approved indications for movement disorders-related chemodenervation	Describes initial dosing and injects common muscle targets with supervision<br><br>Discusses indications for different botulinum toxin formulations<br><br>Formulates initial injection pattern for straightforward cases<br><br>Discusses available guidance techniques (EMG, ultrasound)	Injects common muscle targets without supervision<br><br>Formulates complex injection patterns and appropriately revises injection pattern based on patient response, though may need supervision to inject complex targets<br><br>Uses guidance techniques (EMG, ultrasound) available in their training program with supervision<br><br>Discusses consideration of chemodenervation for off-label indications	Independently injects common and complex targets<br><br>Independently uses guidance techniques (EMG, ultrasound) available in their training program<br><br>Manages side effects of botulinum toxin injection (e.g. dysphagia, weakness, resistance to the toxin formulation)	Serves as an expert in the treatment of complex or refractory patients with botulinum toxin therapy

Comments:

Not Yet Completed Level 1

Not Yet Assessable

**Patient Care 13: Deep Brain Stimulation (DBS) Programming**				
Level 1	Level 2	Level 3	Level 4	Level 5
Discusses basic DBS parameters and the device itself<br><br>Discusses common DBS anatomical targets	Performs initial DBS programming with supervision<br><br>Describes rationale for adjustment of different DBS parameters<br><br>Makes basic DBS adjustments utilizing monopolar montages	Performs initial DBS programming independently<br><br>Adjusts DBS programming using more complex parameters as needed (e.g. bipolar, double bipolar, etc.)<br><br>Understands how side effects relate to anatomy and adjusts accordingly	Programs highly complex and refractory cases (e.g. directional leads, interleaving)<br><br>Discusses emerging and upcoming DBS therapy options	Serves as an expert for efficient and effective DBS programming for a variety of patients

Comments:

Not Yet Completed Level 1

Not Yet Assessable

Medical Knowledge 1: Anatomy, Neurochemistry, Neurophysiology of Movement Disorders				
Level 1	Level 2	Level 3	Level 4	Level 5
Discusses basic basal ganglia (BG) and cerebellar anatomy, network, and function<br><br>Localizes basic movement disorders to specific anatomical regions (e.g. truncal vs. appendicular ataxia, hemiballismus, chorea, Parkinson's disease)<br><br>Discusses basic neuropathology of parkinsonian movement disorders	Discusses anatomical, neurochemical, and pathophysiological hypotheses related to hypokinesia, hyperkinesia, and ataxia<br><br>Describes basic electrophysiologic features of movement disorders (e.g. tremor frequencies, EMG features of myoclonus)<br><br>Localizes more rare movement disorders (e.g. palatal myoclonus)	Describes basic neuropathology of non-parkinsonian movement disorders<br><br>Demonstrates familiarity with pathophysiological hypotheses and data related to movement disorders etiology, diagnosis, and treatment (gut microbiome, specific genetic influences, etc.)<br><br>Discusses proposed pathophysiological and etiological theories for FMD	Discusses nuances of complex and emerging interpretations of BG pathophysiology and electrophysiology<br><br>Discusses pathophysiological hypotheses and data in movement disorders	Engages in scholarly activity on BG or cerebellar physiology<br><br>Engages in scholarly activity on pathophysiological and neurochemical hypotheses in movement disorders

Comments:

Not Yet Completed Level 1

Not Yet Assessable

Systems-Based Practice 1: System Navigation for Patient-Centered Care				
Level 1	Level 2	Level 3	Level 4	Level 5
Demonstrates awareness of existing barriers to care for movement disorders patients<br><br>Appropriately refers to multidisciplinary services (PT, OT, psychology, psychiatry, SW, etc.)<br><br>Demonstrates familiarity of telemedicine in clinical movement disorders	Identifies when a patient faces barriers to care access<br><br>Discusses availability or scarcity of diagnostic or therapeutic resources<br><br>Incorporates telemedicine care as available	Uses available resources to help individual patients overcome **routine** barriers (e.g. transportation, affording a medication)<br><br>Incorporates availability and scarcity of diagnostic and therapeutic resources into treatment decisions<br><br>Communicates with multidisciplinary services to optimize patients' treatment	Uses available resources to help patients access care in **complex** situations (e.g. serious monetary or medical insurance barriers, lack of needed care partner to assist with planning and decision-making)	Contributes to innovations that streamline access to care for underserved or vulnerable movement disorders patient populations

Comments:

Not Yet Completed Level 1

Not Yet Assessable

Practice-Based Learning and Improvement 1: Evidence-Based and Informed Practice				
Level 1	Level 2	Level 3	Level 4	Level 5
Accesses and uses movement disorder literature to address straight-forward clinical questions	Uses knowledge acquired from movement disorder literature to guide formulation<br><br>Critically evaluates scientific literature (e.g. journal club)	Incorporates existing data into clinical practice<br><br>Discusses ongoing research relevant to movement disorders	Critically appraises and incorporates emerging data into patient care, while understanding its limits	Participates in the development of evidence-based clinical guidelines and recommendations

Comments:

Not Yet Completed Level 1

Not Yet Assessable

Practice-Based Learning and Improvement 2: Self-Directed Learning				
Level 1	Level 2	Level 3	Level 4	Level 5
Seeks feedback<br><br>Acknowledges gaps in knowledge and expertise	Incorporates feedback in daily routine<br><br>Uses provided resources to fill supervisor-identified knowledge gaps	Develops an appropriate learning plan based upon clinical experience and feedback<br><br>Reflects on and critiques own performance and develops self-feedback<br><br>Seeks resources to fill self-identified and supervisor-identified knowledge gaps<br><br>Identifies educational resources from professional societies, like MDS, to supplement education	Completes an appropriate learning plan based upon clinical experience and feedback<br><br>Utilizes a variety of resources to fill knowledge gaps<br><br>Uses educational resources from professional societies to inform practice	Counsels others on development and execution of a movement disorders learning plan

Comments:

Not Yet Completed Level 1

Not Yet Assessable

Professionalism 1: Departmental Accountability and Contribution				
Level 1	Level 2	Level 3	Level 4	Level 5
Is prompt and communicates predictable absences in advance	Reliably attends division meetings and activities	Reliably participates and presents during division meetings and activities	Contributes to division-based projects/initiatives (e.g. research, education, community outreach); generates ideas<br><br>Presents or speaks at meetings outside of the division (e.g. Grand Rounds, department meeting, lecture for another department or trainees)	Represents the division/department at national or international meetings<br><br>Leads new initiatives

Comments:

Not Yet Completed Level 1

Not Yet Assessable

Professionalism 2: Clinical Accountability/Conscientiousness				
Level 1	Level 2	Level 3	Level 4	Level 5
Prepares for clinic by reviewing patients in advance<br><br>Responds to prompting about outstanding clinical tasks (e.g. documentation, phone calls)	Completes and documents clinical tasks in a timely and accurate manner **without prompting**, copying all relevant care team members<br><br>Assists other team members if required	Establishes communication goals/timeline with patients as appropriate (phone calls, messages, follow-up)<br><br>Communicates test results to patients promptly and accurately in a format appropriate to the needs of the patient and test result complexity	Follows up with patients proactively as appropriate, even if not patient-initiated<br><br>Communicates proactively with care team members as necessary in unanticipated situations (hospitalizations, etc.)	Develops systems and practices to help self and others function more effectively as clinicians
☐ ☐	☐ ☐	☐ ☐	☐ ☐	☐

Comments:

Not Yet Completed Level 1 ☐

Not Yet Assessable ☐

Interpersonal and Communication Skills 1: Communication within Team				
Level 1	Level 2	Level 3	Level 4	Level 5
Interacts with entire medical team respectfully<br><br>Communicates using language that values all team members	Demonstrates positive relationship with entire care team	Constructively navigates basic conflicts independently<br><br>Initiates debriefing and conflict resolution as needed	Navigates complex conflicts without compromising professional behavior	Role models conflict management including self-reflection

Comments:

Not Yet Completed Level 1

Not Yet Assessable

Interpersonal and Communication Skills 2: Communication with Patient and Family				
Level 1	Level 2	Level 3	Level 4	Level 5
Demonstrates a positive relationship with patient<br><br>Communicates with cultural sensitivity and respect using clear language	Demonstrates a positive relationship with patient and family; establishes rapport in challenging situations<br><br>Practices active listening during patient interview; utilizes individualized approach; probes for patient understanding<br><br>Counsels patients on risks/benefits of treatment, including limitations of available data	Solves basic conflicts with patients through interpersonal skills<br><br>Involves patient and family as partners in clinical decision-making<br><br>Initiates difficult discussions with compassion	Navigates complex psychosocial dilemmas with patient and family<br><br>Effectively leads complex discussions including prognosis, advanced care needs, genetic testing, goals of care, controversies, uncertainties, functional diagnoses, and ongoing research/clinical trials	Role models navigation of complex patient interactions, and models self-reflection to further develop interpersonal and communication skills<br><br>Role models optimizing patient communication (e.g. develops patient education materials)

Comments:

Not Yet Completed Level 1

Not Yet Assessable

Interpersonal and Communication Skills 3: Communication with Other Providers				
Level 1	Level 2	Level 3	Level 4	Level 5
Clearly documents history and appropriate neurologic examination	Documents clear clinical reasoning/formulation<br><br>Ensures all relevant providers have access to documentation	Initiates direct communication with relevant clinical providers as needed<br><br>Expresses divergent opinions respectfully	Manages discussions between relevant concurrent providers with follow through<br><br>Forms collaborative relationship with other providers to maximize patient care	Serves as a focal point of contact between medical providers to maximize patient care<br><br>Expertly manages disparate opinions between providers

Comments:

Not Yet Completed Level 1

Not Yet Assessable

Appendix for Fellowships With Specialty Training

Practice-Based Learning and Improvement 3: Scholarly Work				
Level 1	Level 2	Level 3	Level 4	Level 5
Determines field/methods of interest (e.g. bench research, translational research, clinical trials, QI, med education, etc.)<br><br>Works with appropriate teams to learn about the field<br><br>Establishes a mentorship relationship	Finalizes project protocol, outline, and timeline including goals<br><br>Begins data collection	Demonstrates clear progress along planned project timeline and protocol<br><br>Completes data collection and begins analysis	Completes and interprets data based on analysis<br><br>Develops and submits manuscript to peer reviewed journal or poster/abstract to conference or other scholarly avenue	Publishes article in peer reviewed journal<br><br>Mentors and supervises junior researchers in movement disorders scholarship<br><br>Takes a lead role in writing and submitting a research grant

Comments:

Not Yet Completed Level 1

Not Yet Assessable
